# Game-Based Learning and Service-Learning to Teach Inclusive Education in Higher Education

**DOI:** 10.3390/ijerph20043285

**Published:** 2023-02-13

**Authors:** José M. Rodríguez-Ferrer, Ana Manzano-León, José M. Aguilar-Parra

**Affiliations:** 1 Department of Psychology, University of Jaén, 23071 Jaén, Spain; 2 Health Research Centre, Department of Psychology, University of Almería, 04120 Almería, Spain

**Keywords:** game-based learning, service-learning, inclusive education, engagement, flow, higher education

## Abstract

This study evaluates the impact of game-based learning (GBL) and Service-Learning on the flow and engagement of teacher education students. A quasi-experimental between-group comparison design with pre-test and post-test measures was conducted with a sample of 113 students majoring in childhood education. The results indicate that the experimental group statistically significantly improved their flow and engagement scores compared to the control group. It is concluded that the GBL and SL methodology in initial teacher training allows students to learn about inclusive education in a motivating way and to design different strategies and resources that they will be able to use in their professional future.

## 1. Introduction

Nowadays, more training is needed for students to participate as active citizens [1]. Education, including the university stage, can play a leading role in training to transform a more inclusive society. In this educational model, the aim is that students are trained in curricular content and, simultaneously, they are oriented towards training in competencies for living sustainably in the personal, work and community contexts [2,3].

Service-Learning (SL) is a methodology that combines academic learning and training for active citizenship [4]. This is achieved in a single well-articulated project, in which students are trained, getting involved in real needs of the context in order to improve it [5]. SL is a methodology oriented to education for citizenship, inspired by active pedagogies and compatible with other educational strategies such as cooperative learning [6] and project-based learning [7]. Recent research shows that SL can be a methodology that reports great benefits in university students, and it is linked to an improvement in the quality of learning [8,9,10,11]. The benefits highlighted include the development of leadership competencies, the promotion of generosity, helpfulness and empathy behaviors [12], the improvement of self-esteem and development of social skills [13] and the acquisition of significant learning [14]. Teacher training should offer theory and practices based on proper child development so that in their professional future they can respond to the educational needs of their classrooms [15]. Among the didactic strategies in early childhood education, game-based learning (GBL) [16,17] stands out, especially in the early stages of childhood. Play encourages exploration of the world and problem solving. In addition, it facilitates children to work on executive functions in an intrinsically fun manner [18]. Currently, children have fewer play experiences due to sociocultural barriers, such as excessive seriousness in their daily routines, less family time for leisure and sedentary lifestyles [19]. Therefore, there is an urgent need for teacher training to encourage play in children in early childhood education, and thus create an active learning environment [20]. Various research shows how the use of playful learning strategies can also be highly motivating for university students and create meaningful learning experiences [21,22,23,24].

Another noteworthy feature of GBL and SL is their ability to generate engagement and learning [25,26,27]. Educational engagement is a key element in university education and is positively related to learning outcomes [3,28,29,30]. Educational engagement is defined as students’ immersion towards the activities performed in class, and the higher this engagement, the more engaged students will be with the tasks designed for learning [31,32]. To achieve this, it is mentioned that activities and challenges must be designed so that students consider them achievable, and in turn present a challenge for them, as well as present them with clearly defined goals and objectives, direct feedback and that they have the feeling of having some control over the tasks they perform [33].

Furthermore, Flow theory has been widely related to playful learning strategies [34,35,36,37,38,39], due to the enjoyment and motivational capacity of games. Csikszentmihhalyi [40] mentions the importance of creating an optimal experience for students, where they can enjoy the activity and be immersed in it. Sillaots [41] shows that the concentration and commitment of students who performed gamified activities was greater than in those students who took a master class, demonstrating that gamified activities allowed greater creativity and that the game elements acted as a motivational factor. These results coincide with other research that mentions the potential of the game to achieve high levels of immersion, understanding of concepts and learning.

The purpose of this study is to highlight the engagement and flow of students majoring in childhood education through a SL and GBL methodology. Several studies have investigated the impact of SL on engagement [42,43,44]. However, little research has addressed its combined use with GBL in teacher training [45,46]. The following objective has been set: to evaluate the impact on the flow and engagement of the playful SL in university students in comparison to directive education.

## 2. Materials and Methods

A non-probabilistic quantitative quasi-experimental methodology was used, with an experimental group and a control group, with pre-intervention and post-intervention measurement of flow and perceived engagement. The quasi-experimental designs are characterized by the manipulation of at least one independent variable (Teaching–learning style, comparing traditional practices with SL practices and game creation) and the measurement of at least one dependent variable (flow and engagement), although the homogeneity of the groups is not guaranteed, since they were already formed prior to the intervention [47].

The study was conducted in accordance with the Declaration of Helsinki and approved by Academic Planning and Teaching Committee of the University of Almería (22_23_1_01C).

### 2.1. Participants

All participants were second-year students of the Degree in Early Childhood Education enrolled at the University of Almería during the 2021/2022 academic year.

An intentional non-probabilistic sampling method was performed. The inclusion criteria were a participation in the practice sessions greater than 75% and the delivery of informed consent for participation in the study.

From this population, a sample of 55 students who participated in the experimental group, 5 men and 50 women, with a mean age of 21.24 (SD = 2.46), and 58 students who participated in the control group, 9 men and 49 women, with a mean age of 20.92 (SD = 2.40) were collected. The reason why the sample is predominantly composed of women is because the university degrees in the area of Education are chosen mainly by women [48].

### 2.2. Instruments

The assessment instruments used were as follows:

Brief Inventory of Optimal Experiences [49]. Is a Likert-type inventory with 5 response possibilities. The response possibilities range from 1 “Strongly disagree” to 5 “Strongly agree”. The flow variable is obtained in a summative way from each of the 9 items that compose the inventory. The psychometric properties of the instrument were not adequate (*AGFI* = 0.90; *GFI* = 0.94, *TLI* = 0.95; *CFI* = 0.96; *RMSR* = 0.05; *RMSEA* = 0.07) and Cronbach’s alpha was 0.864.

The Classroom Engagement Inventory [50], adapted to the Spanish population [32]. The questionnaire is composed of 24 items, which are divided into five factors: Affective Engagement, Compliance, Behavior Engagement, Cognitive Engagement and Disengagement. Items have five response options from Totally Agree to Strongly Disagree, except for the Cognitive Engagement factor which is divided into seven response options. This questionnaire has been validated by a factor analysis and an internal consistency analysis where the values of Cronbach’s alpha are higher than 0.80.

### 2.3. Procedure

First, the control group and the experimental group were divided by their natural classroom context, i.e., once the two teachers of the subject agreed to participate in the research, both groups were tested for equivalence in the variables studied by means of a pre-test.

Both groups participated in the practices of a second-year subject on inclusive education. These practices were developed in eight two-hour sessions, respecting the hygienic measures proposed by COVID-19, and accepting online assistance in case any student had to be confined at home.

In the control group, the teacher taught the practices with a directive methodology supported by PowerPoint presentations. The students had to deliver a project in groups of 4 to 6 people. This project consisted of making a PowerPoint of the subject content and making the presentation. This project had a maximum score of three points on the final grade.

In the experimental group, the teacher explained the practical content with a presentation. A playful SL project was proposed, which would also count for up to three points of the final grade. In the playful SL, groups of 4 to 6 students had to design and manufacture games and playful material to donate to educational centers in the province.

The objectives of the application of this methodology were for students to be able to (a) describe and differentiate special needs; (b) integrate information from description, assessment, and intervention in inclusive education; (c) design games to work on the different areas that may be affected by special needs.

**Figure 1:**
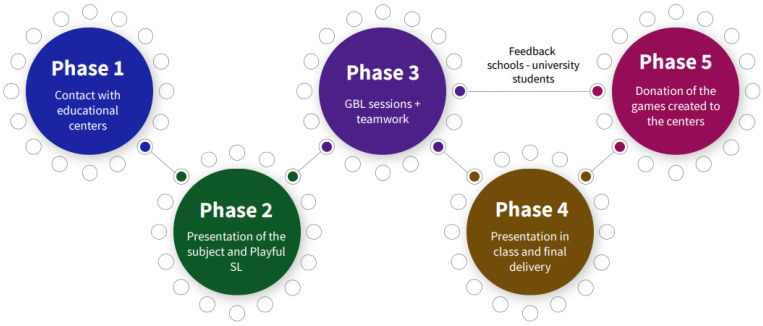
Phases of Playful Service-Learning.

The methodology was carried out in the following phases (See Figure 1).

For the development of this Service-Learning, an appeal was made through social networks and email to different centers in the province with the information of the project and the request for collaboration. This collaboration consisted of the fact that teachers had to develop the case of a child with special needs, in a text of two pages maximum. These cases were reviewed, removing any sensitive information and, if necessary, some extra feature was developed to complete the case.

**Figure 2:**
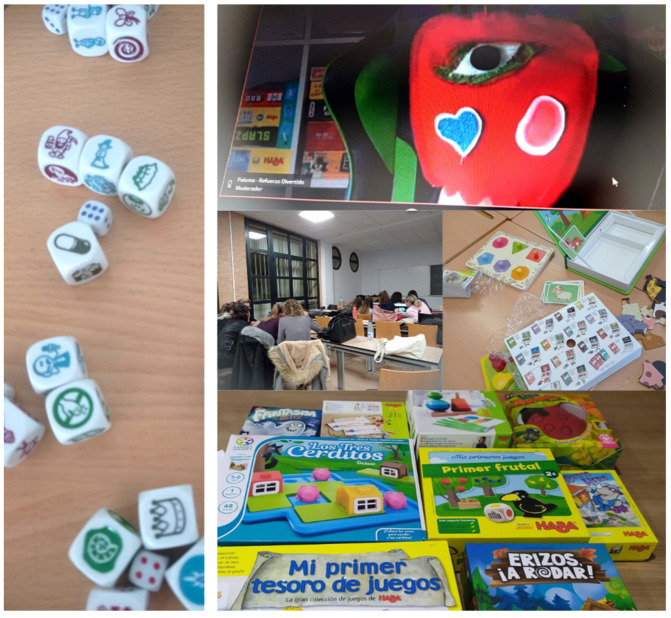
Examples of games and resources learned within the Playful SL.

To train students on play-based learning and using different playful resources, demonstration sessions and teamwork were held, where the teacher showed different games and resources in the classroom (See Figure 2).

These cases were randomly distributed to the student groups. In the practice sessions, different didactic strategies were used to work specifically with students with special needs or through the universal design for learning [51].

**Figure 3:**
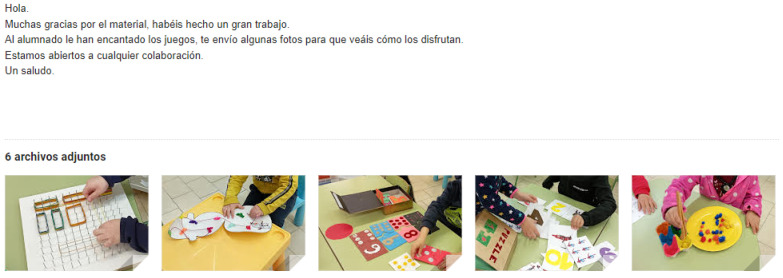
Example of school feedback. Note. Translation: Hello, thank you very much for the material, you have done a great job. The students have loved the games, I send you some photos so you can see how they enjoy them. We are open to any collaboration. Greetings.

When the students finished the games, they made a presentation to work on the students’ oral skills, a key aspect for their performance as future teachers [52]. After the presentation, these materials were donated to the participating schools to encourage the participation of students in the community [53]. Finally, schools offered feedback on the games (See Figure 3).

On the other hand, the control group carried out the practices of a subject of a 2nd year degree of Early Childhood Education with a more traditional methodology. Groups of 4 to 6 students were formed who had to make a PowerPoint presentation on the subject; they worked on the subject and presented it on the last day of practice. The practices of this group had the same duration, eight sessions of two hours.

### 2.4. Data Analysis

First, the direct scores of the assessments performed were calculated to answer the research objective. Next, a Student’s *t*-test was performed to check whether the groups were equivalent. Finally, an rmANOVA test was performed for each of the study variables and the corresponding post hoc tests were carried out. Bonferroni was used as the adjustment statistic for the statistical tests. All analyses were performed with SPSS version 25 (IBM Corporation, Armonk, NY, United States).

## 3. Results

First, all mean and standard deviations have been reported in Table 1. An analysis of the difference in pre-test means between the control group and the experimental group of the studied variables has been carried out. In Table 2, no statistically significant differences were observed between them in any of the variables analyzed during the pre-test, so it can be considered that they were equivalent groups to each other.

To know the effect of the playful SL methodology, an rmANOVA test has been performed for each variable under study, the reported statistic is Wilks’ Lambda: Flow (F = 62.35, *p* < 0.001); Compliance (F = 100.98, *p* <.001); Affective engagement (F = 29.41, *p* < 0.001); Behavior engagement (F = 111.00, *p* < 0.001); Cognitive engagement (F = 111.00, *p* < 0.001) and Disengagement (F = 51.15, *p* < 0.001). As can be seen in all variables, statistically significant differences have been found. In the Table 3, the inter-subject and intra-subject tests have been reported with the corresponding post hoc tests.

In the intra-subject tests, there were changes in all variables in favor of the experimental group, except in Disengagement in favor of the control group; therefore, they have a lower commitment to the practices. As for the inter-subject tests, there are changes in the experimental group in all variables, while the control group only had a change in the variable Disengagement, increasing it after the application of the traditional methodology. Although the experimental group has decreased its scores in this variable in the post-test scores compared to the pre-test, the control group has undergone the reverse change and increased its scores in the post-test scores.

## 4. Discussion and Conclusions

The aim of this research was to evaluate the commitment and flow of teacher training students towards a playful SL methodology. This objective is based on the need to promote innovative learning practices for university students, especially those who in their future work will educate children, so consequently they need to learn different strategies to be able to implement them.

There is a need to teach practical content and to be able to assess the use of real cases and simulations in higher education, with the purpose that students feel more prepared for their professional performance and feel motivated [54,55,56,57]. SL can favor this type of practice, by learning with real cases, and it also provides a benefit to the community thanks to the work done by the students [58].

It has been observed that with this playful SL methodology, a greater engagement and flow has been achieved than with a traditional methodology. The results obtained in this research coincide with previous research [6,59,60] that shows that this methodology can be very motivating for university students. It is highlighted that the fact of implementing a methodological design that poses achievable challenges for students and where the teacher guides the learning process, giving feedback in each session, can be key elements to promote flow among university students.

Although the results reveal promising developments in playful SL research in teacher education, there are several limitations. First, the present study is limited by the relatively small sample size. Larger samples are needed to generalize the results, and future research could even assess whether there are differences depending on whether the students are pursuing a Bachelor’s or Master’s degree. Another limitation is the method of sample selection, since, due to the collection of the data in the natural context of the classroom, it was not possible to perform analyses according to gender or age, since the groups were mostly composed of women between 20 and 25 years of age. In addition, the program was applied during a four-month course, so it was not possible to determine the long-term effect of this methodology. Future research could focus on longitudinal studies on playful SL, which contemplate the combined study with other variables such as academic performance, anxiety towards evaluation or classroom climate, as well as evaluating the effectiveness of the methodology for other agents of the educational community.

It is concluded that the playful SL methodology in teacher training allows for the learning of valuable content in a motivating way for students and designing different games that they can use in their professional future. Furthermore, the games and materials produced during the practices have a real utility in the educational community.

## Figures and Tables

**Table 1:** Means and standard deviations of the variables studied in the control and experimental groups.

	Control Group				Experimental Group			
Variables	Pre-M	DT	Post-M	DT	Pre-M	DT	Post-M	DT
Flow	30.07	5.30	30.74	5.73	30.55	4.46	35.05	3.41
C	17.95	3.41	18.91	3.23	18.14	2.13	22.41	2.32
A.E.	16.83	3.38	16.76	2.90	17.02	3.08	19.80	2.20
B.E.	16.31	3.29	16.91	3.07	16.45	3.05	20.30	2.54
C.E.	32.37	6.56	32.24	5.84	33.02	6.38	42.20	6.78
D	6.36	2.55	7.89	2.80	6.13	2.77	4.69	1.84

Note: C: Compliance; A.E.: Affective Engagement; B.E.: Behavior Engagement; C.E.: Cognitive Engagement; D: Disengagement.

**Table 2:** Student’s *t*-tests for independent samples with the pre-test scores of the variables under study.

Variable	*t*	*p*
Flow	0.505	0.615
Compliance	0.365	0.715
Affective engagement	0.315	0.753
Behavior engagement	0.231	0.817
Cognitive engagement	0.532	0.596
Disengagement	−0.478	0.633

**Table 3:** Inter-subject and intra-subject tests with post hoc tests.

	Intra			Inter		
Variable	F	*p*	Post Hoc	F	*p*	Post Hoc
Flow	34.60	***	Exp ***	7.95	0.006	Exp ***
Compliance	41.02	***	Exp ***	16.27	***	Exp ***
Affective engagement	32.27	***	Exp ***	10.83	0.001	Exp ***
Behavior engagement	150.40	***	Exp ***	176.95	***	Exp ***
Cognitive engagement	1221.93	***	Exp ***	68.29	***	Exp ***
Disengagement	51.15	***	Cont ***	16.94	***	Exp *** Cont ***

*** = *p* < 0.001; Exp = Experimental; Cont = Control.

## Data Availability

Data are available at the request of the corresponding author.
